# Determining the green technology innovation accelator and natural resources towards decarbonization for the EU countries: evidence from MMQR

**DOI:** 10.1007/s11356-024-32302-4

**Published:** 2024-02-15

**Authors:** Ibrahim Cutcu, Dilek Cil, Cigdem Karis, Sinem Kocak

**Affiliations:** 1 Department of Economics, Hasan Kalyoncu University, Gaziantep, Türkiye; 2 Department of Travel Tourism and Entertainment Services, Trabzon University, Trabzon, Türkiye; 3 Department of Finance Banking and Insurance, Trabzon University, Trabzon, Türkiye; 4 Independent Researcher, Independent Researcher, Trabzon, Türkiye

**Keywords:** Green technology innovation, Natural resources, Ecological footprint, Environmental tax, Financial development

## Abstract

Gearing up for green technology innovation (GTI) and natural resources has become even more important in the transition to a zero-emission life, a green economy, and sustainable development goals. This attempt has become a situation that needs to be overpowered much sooner by the European countries, which have encountered challenges in many ways, especially regarding natural resources, energy supply, and the climate crisis. In this vein, the current study follows the novel, robust Method of Moment Quantile-Regression (MM-QR), which successfully yields heterogeneous information structure across quantiles, to examine the determinants of GTI for 15 EU countries over the period of 2003–2018. MM-QR estimation results indicate that the determinants of green technology innovation are heterogeneous across the EU countries. While green growth (GG) has an adverse impact on GTI in middle- and high-GTI countries, the effect of ecological footprint on GTI is positive for countries in the highest-GTI countries. The positive effects of financial development (FD) on GTI are revealed for all countries. Remarkably, environmental taxes have an adverse and positive influence on GTI in the lowest and highest quantile countries, respectively. Finally, renewable energy and greenfield FDI have no effect on GTI. Governments can promote GTI by providing financial resources, in the most immaculate way, to firms that engage in green technology projects, as well as by encouraging these through environmental taxes.

## Introduction

Recent droughts, floods, shortages of water, and rising mean temperatures and sea levels have made climate change an existential menace to the world. This challenge has become a more complex reality that the whole world must face. Today, changes in climate have attracted all the attention on a global scale but have exhibited no equal distribution worldwide. Unfortunately, current concerns have been exacerbated. Substantial factors, together with the pandemic and just after Russia’s invasion of Ukraine, have contributed to the chaos, especially in the energy markets. The diminution of the negative repercussions of this transformation has become the only focus of both short- and long-term projections. However, all breakthroughs in the future may also mean an opportunity to form a new global energy economy. Anyway, the growing global energy economy is expected to be mostly based on the green transition of the utilization of non-fossil sources, technologically advanced technologies, and the generation process of green energy. The evolution of the global energy system has been iterating away from the preference for fossil fuels and towards low-carbon technologies. The most indispensable argument for green transition is seen as green technology innovation (GTI), known as environment-friendly technology. Empowering technology for a green economy is more vital than ever. However, green technologies that are now essential for the global energy transformation necessary to adhere to the 1.5 °C Paris Agreement would need to be implemented at considerably quicker rates and on a greater scale (IRENA 2023).

As outlined in the International Energy Agency (IEA) report (2023), the advent of a new era of clean technology industries is the most significant indicator of this transition. Besides, overcoming energy and climate goals requires the strategic scaling up of clean energy technologies (IEA 2020). The potential in the new greener technologies such as renewable energy technologies, innovation in electrification, hydrogen, hydrogen-related fuels, bioenergy and carbon capture, utilization and storage for transport and industry, and low-carbon power generation inevitability becomes the functionally top focus of reaching net-zero ambitions. With its unique qualities and standards, GTI is an effective way to truly address ecological and environmental issues. Actualizing technological innovation in much more fuel solutions for the future is an imperative part of green transitions. It is also an essential technological pathway to support green development (Yi et al. 2019).

GTI, on the other hand, may be a remedy to the environmental issue since it aids in combining sustainable economic growth with improved environmental management (Sharif et al. 2023; Kocak 2023; Cinar and Yilmazer 2021). By effectively allocating and utilizing energy resources, enhancing green innovation will increase production and ultimately reduce carbon emissions (Guo et al. 2018a, b; Sharif et al. 2022). Although significant advancements have been made, the need for cleaner technology is still young. Moreover, greener technology is already ready to replace traditional energy systems, making it a cornerstone of the future transition. GTI is seen as the key to tackling the economy-environment problem since it enhances operational effectiveness while lowering adverse impacts on the environment (Li et al. 2021).

However, this is not the way to go by GTI alone, so determining the underlying factors affecting GTI is very important in this respect. GTI holds some environmental factors in itself. Factors for the environment, such as promoting the use of renewable energy, environmental taxes (Popp 2012; Johnstone et al. 2008; Li and Lin 2016), and foreign direct investment (Behera and Sethi 2022), have the presence of intense environmental pollution and have potent to be the driving forces of GTI. According to Edenhofer et al. (2011) and Bel and Joseph (2018), one of the clearest indicators of catching the change in GTI is the share of energy from renewable sources.

Environmental taxes are another policy tool that the government might favor to promote GTI. There is a positive function of environmental tax to promote GTI. Environmental taxes have been an encouraging tool in the production of innovative activities in cleaner technologies (Acemoglu et al., 2016). Particularly, carbon pricing taxes are also significant stimulants of low-carbon, energy-efficient innovation (WB 2016). Confronting higher tax-inclusive fuel expenses is a pioneering power that makes firms more innovative in clean (and less filthy) technology (Aghion et al. 2016). As to greenfield investment, it has a broad range of larger and more complicated meanings, comprising investments in environmental, social, and governance initiatives that are intended to promote long-term sustainable development goals (Inderst et al. 2012 ; Kwilinski et al. 2023). It plays an effective role in fostering GTI by positively affecting specialization (Castellani et al. 2022). According to Khan et al. (2022), it is vital to promote greenfield investment for GTI, as greenfield investment is needed for the enforcement of green innovation. Green foreign direct investment plays an effective role in fostering GTI. Similarly, therefore, foreign direct investments are another effective way of promoting the transfer of technology and innovations from the mother countries to the host countries through the technology spillover effect (Xu et al. 2021a, b; Zeraibi et al. 2023). In addition, it is one of the most desirable and inevitable situations in which multinational companies will gain importance in the green FDI investments of the future, given the current incentives provided for the adoption of practices and technology that will increase the environmental quality of their production processes. In this context, leading companies that have already made the breakthrough will be able to gain a competitive advantage by taking steps to transform their operational processes into environmentally friendly technologies. Specifically, due to the increasing enthusiasm of green FDI investors to invest in companies that include GTI investments in their operational processes, these companies will be able to expand their market shares internationally by gaining cost advantages. However, as local companies accept a new green technology through green FDI and the speed of implementing this innovation into their processes increases, the rate of spread of green technology will also be positively affected (World Bank 2008; Popp 2009; Popp, 2010). Therefore, it is expected to drive the GTI.

Another potential channel that can be used for green technology innovation is green growth. The World Bank suggests that “green growth” involves achieving resource efficiency, cleanliness, and resilience in economic expansion without impeding its pace. One of the most effective ways to achieve this is to increase the number of applications and investments in green technology innovations, both in quantity and quality. Thus, green growth becomes an indispensable feedback factor for green technology innovations (Hallegatte et al. 2012; Toman 2012; Fay 2012; Rosenbaum 2017).

On the other hand, carbon emissions and ecological footprints intrinsically have the potential to be one of the factors that can influence GTI. Being an indicator of how much the earth has been polluted, the ecological footprint necessitates the progress of greener technologies. As the ecological footprint increases, it is natural that the need for innovations in green technologies increases. Specifically, accelerating energy demand causes both carbon emissions and ecological footprints to emerge as serious environmental problems today. In such a situation, it is clear that there is a need for new technologies to soften the increasing environmental degradation. Alias, it would be a very correct approach to expect that increasing emission levels should bring about a rising need for GTIs. Governments are expected to formulate policies to encourage innovations in green technology to tackle the resulting increase in either of these two important environmental indicators.

One of the underpinning channels for facilitating GTI is the existence of a well-developed financial system. Green finance is essential for funding clean energy and renewable energy projects that reduce carbon emissions and their harmful effects on the environment’s sustainability and human health. It incorporates considerations of sustainability into financial choices. Therefore, it is anticipated that by supporting climate-neutral, energy-efficient, and resource-efficient technologies, these sustainability reflections on green finance will enhance environmental and sustainability considerations (Madaleno et al. 2022; Hu and Zhang 2023). Bankrolling greener technologies in the framework of the relationship between finance and climate change is now at the core of the attempts at decarbonization. As outlined in  De Haas and Popov (2023), firstly, stock markets can help promote the build-up of greener technologies by contaminating industries. On the other hand, having deeper stock markets is one of the most intensive functions of the development and deployment of the innovation process. It also has a key role in assisting more patenting of green innovations in carbon-intensive sectors, leading to lower carbon emissions per unit of output.

However, there also exists a standpoint in the investigations that a strengthened climate change policy can reduce output by increasing alternative energy efficiency, and as a result, this may reduce the motivation for GTI (Park and Funk 2020).

Cleaner tech is a fundamental constituent of the agenda of zero-emissions life and will pursue to perform an integral role in going green. Accelerating innovation in more cleaner technology is vital to the global climate strategy (Li et al. 2023a, b). Because of the following reasons, this attempt may now be more indispensable for the EU, which, for more than two decades, has been leading the struggle against climate change (European Commission 2018; Teixidó et al. 2019). Particularly, the EU desires more in-depth strategies for a shared sense of urgency regarding greener problems (European Commission 2020). First of all, the EU had started actions at an earlier date, emphasizing the urgency of climate change mitigation measures by setting up its own Paris Agreement Compatible (PAC) energy roadmap for renewables, energy efficiency, and carbon reduction. For this, the EU takes steps towards net-zero emissions by 2040 instead of 2050 and to shift 100% of the energy supply to renewables until 2040 while concentrating on the climate emergency (Chen et al. 2023). The goal of implementing these initiatives in 2040 instead of 2050 is important and also the first indicator of how seriously the EU takes the transition to low-carbon.

Secondly, renewable energy source-related technologies have been at the forefront of the EU (Conti et al. 2018). The EU has also carried out policy-oriented actions to deepen its bilateral ties through cooperation with its strategic partners, such as deploying low-carbon technologies, converging relevant carbon markets, and cooperating closely on carbon pricing. Through the EU Emissions Trading System (EU ETS), the EU formed a series of associations to promote and invest in innovations in low-carbon technologies (European Commission 2018). This step is a reminder that the EU is keen on an innovation-driven climate strategy. These collaborations and policy actions highlight that innovation in low-carbon technologies has always been one of the top priorities for the EU. More recently, the European Commission prioritized the Net-Zero Industry Act (NZIA). By speeding the development and production of net-zero technologies, the Act also aims at minimizing the risk of replacing our dependence on Russian fossil fuels with other strategic dependencies that could impede our access to vital technologies and components for the green transition (European Commission 2023).

These steps were, of course, just before the EU experienced a much more important process. Unfortunately, especially after these attempts, and thirdly, the expedition to decarbonize the EU has faced challenges far more formidable than any other. With the coming out of the Ukraine war, conducting the challenging effects based on the supply chain and energy collaboration has just come a bit harder for the EU. Pressure on the EU energy markets has freshened. These unfavorables have joined in the rapidly advancing climate concern. This development brought the EU to a critical moment of new rapid energy attempts and to the brink of new breakthroughs. For the EU, now that it is not a latecomer region, faster progress in technology is much needed.

**Fig. 1:**
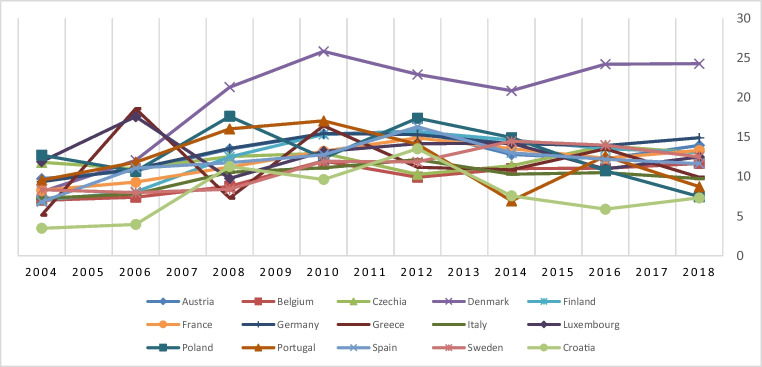
Number of patents in environment-related technologies in the EU by OECD Database

The last one is emphasized by Aghion et al. (2022). They argued that, compared to a few chosen counterparts, the EU has a lower long-term rate of patenting “green” inventions. Furthermore, they claim significant heterogeneity within the EU states: although one-third of them have fewer than one “green” patent per million people annually, several are world leaders in patented “green” innovations. For example, the Danish government has established the lofty aim of making Denmark totally a global hub for innovation. What is particularly noteworthy is that Denmark, including countries within the EU, dominates the development of green technology innovation worldwide (see Fig. 1). Certain countries in particular, such as Germany, Austria, Finland, Sweden, and France, show an upward trend in the development of green technology innovation, although not as much as Denmark. Croatia, Poland, Italy, and Portugal are the countries with lower levels of green technology innovation compared to others in this group. In fact, Croatia and Poland in particular have been following a downward trend with instability in recent years. They also underlined that, contrary to what its size and riches might imply, the EU has thus far made less of a contribution to advancing innovative green technology. The average EU member state has lagged behind in this process noticeably. However, after all these developments, the technological innovation of cleaner energy, which is already essential for the EU, has become even more necessary with all these difficulties to reach the emission reduction targets sooner. If so, the EU may be expected to integrate technologies into green energy faster than in the past. It seems that the need for the use of low-carbon technologies has the potential to be more urgent for the region now. Therefore, all the motivations mentioned are just a few of the key reasons to focus on the EU as a very important example in this article.

In addition, the lack of papers focusing on the determinants of GTI in the literature is remarkable. The existing body of literature is growing but still narrow. Also, the topic is old (Bel and Joseph 2018; Cinar and Yilmazer 2021; Lou et al., 2021; Lv et al. 2021; Behera and Sethi 2022; Sharif et al. 2023), but empirically new for the EU. Based on a dataset of the EU-15 countries from 2003 to 2018, this paper aims to highlight the potential determinants of GTIs such as environmental taxes, renewable energy, financial development, green FDI, ecological footprint, and green growth for EU countries to inspire accelerating the process of the journey to net-zero emissions. Especially by demystifying the view of Aghion et al. (2022), the paper also seeks another response to the query: are the determinants of GTI in different EU countries diverse from each other, namely heterogeneous? While shed lighting greener innovation in the propping of climate and energy targets, thus, the major opportunities for the EU countries in the process of clean energy transition guidance can be identified.

Considering all these points of view, this research emphasizes the following three motivations that the literature offers: first, while the present analysis is one of the pioneering attempts to empirically examine the determinants of GTI, especially for the sample of the EU, it also aims to expand the nascent literature. Secondly, in addition to tools such as environmental taxes, renewable energy, and financial developments frequently debated in the literature (Li et al. 2023a, b), unfortunately, numerous papers investigating the role of green FDI, ecological footprint, and green growth on green innovation are not available in the literature. So, the most attention-grabbing dataset of novel environmental indicators consisting of green growth, ecological footprint, and green foreign direct investments is tackled in the present analysis with the previously mentioned variables. In this sense, the scope of the study has been expanded, and the role of these variables on GTI has been examined in detail. Because these indicators are gaining popularity as alternative routes to struggle environmentally, they are expected to prompt innovation activities in green pathways. Moreover, the paper attempts to put forth policy clues for action to realize the desired targets of the EU by generating better empirical evidence. Thirdly, and finally, using a quantile-based approach is the most proper econometric approach to reveal the evidence for the above-mentioned research question as well as the other conditional mean approaches. Closest to our objective, we utilized both the novel and robust approach of the Method of Moment Quantile-Regression (MM-QR) because of its success in revealing the heterogeneous information structure across quantiles, which numerous other econometrics approaches are unable to achive. MM-QR is the best method for this analysis because it lets us see how the variables in the new dataset change the levels of GTI at different quantiles. So, the estimation procedure is quite successful in catching heterogeneous effects across the quantiles. Previous research rarely highlighted the presence of heterogeneity or distributional variation in the factors of GTI. To demonstrate clearly, we make use of the advantages of the unique MM-QR technique. Thus, it underlines heterogeneous factors necessitating primary attention within the EU countries for GTI. To sum up, this study aims to advance the aforementioned gaps in the development of the scant literature about the EU. It is also among the first to experimentally examine the determinants of GTI, especially for the EU sample. Second, in addition to weapons like environmental taxes, renewable energy, and financial developments that are regularly discussed in the literature, the analysis addresses the most significant new environmental indicators, such as green growth, ecological footprint, and green foreign direct investments. Third, previous studies have not looked into EU countries. Fourth, the data in the green panel is GTI. Green patents can serve as an accurate gauge of R&D spending and reflect shifts in the sector. Innovation-related environmental activities can be measured in terms of number and quality. Lastly, sophisticated econometric methods like the homogeneity test, cross-sectional dependence test, PANIC unit root test, and Durbin-Hausman cointegration method were used to provide reliable results. Moreover, the MM-QR methodology was employed to evaluate the impact of the designated variables.

By examining the determinants of GTI, this research aims to dilate the available evidence, focusing on the EU countries. To do that, the paper is assembled under the following four main headings, respectively: literature, methodology and data, results section, and lastly, the conclusion part.

## Literature review

Previous research has given great attention to the determinants of environmental pollution as a result of the increasingly severe impacts of environmental degradation on economic performance and natural life. GTI provides a double benefit by promoting both economic and environmental health. Since the 1990s, utilization of green technology has been steadily expanding worldwide, primarily in industrialized countries, establishing a green future (Behera and Sethi 2022). GTI is a factor that has lately attracted attention in the environmental economics literature as a factor affecting environmental quality. With the continued progress of GTI, an increasing number of politicians and researchers have noticed the significance of technological innovation for environmental quality by reducing pollutant emissions and ecological footprints (Wang et al. 2021; Jian and Afshan 2022; Koseoglu et al. 2022; Metawa et al. 2022; Sharif et al. 2022; Hou et al. 2023; Javed et al. 2023; Liu et al. 2023). GTI, targeted at minimizing the effects of economic activity on climate change, is an effective instrument for transitioning to a low-carbon economy (Bel and Joseph 2018). Green panel data is a well-known indicator for measuring GTI. Various indicators, such as patent and trademark applications, the number of research studies, technical cooperation awards, and government R&D investment, have been used in the literature to reflect technological innovation. However, while these variables are relevant to technological innovation, they are not relevant to environmental innovation. This could not indicate scientific development in environmentally friendly Technologies (Chen and Lee 2020; Sinha et al. 2020; Adebayo et al. 2022). Researchers recently used green patent data to measure GTI (Shen et al. 2021; Yu et al. 2021; Huang et al. 2022; Yang et al. 2022; Bai et al. 2023; Hu et al. 2023; Kocak 2023). Patents can reflect changes in the industry and are a tangible measure of research and development expenditures. Environmental activities related to innovation can both reflect quantity and quality. Green patent practices are increasingly being adopted as a result of public environmental policy (Cinar and Yilmazer 2021). Green patents are used as a better proxy for GTI in this study.

GTI can improve energy technologies, processes, and products and help reduce the waste of raw materials and environmental degradation (Braun and Wield 1994; Chen et al. 2006; Huang et al. 2022, 2019; Sandberg et al. 2019). In order to reduce the use of natural resources, lessen ecological harm, and increase the effectiveness of resource allocation, green innovation offers new goods, procedures, services, and market solutions. As a result, it becomes the motivation behind promoting and implementing ways for sustainable economic development (Huang et al. 2022). Additionally, GTI lowers energy consumption, which then promotes sustainable development (Zhou et al. 2017; Zou et al. 2017). According to Lund (2007), there are three approaches to accomplishing sustainable development through technological innovation in the energy sector: energy savings on the demand side, increased energy production efficiency, and the substitution of fossil fuels with a variety of renewable energy sources. Hu (2023) argues that the effects of technical innovation and energy efficiency improvement are powerful mechanisms.

GTI has attracted the interest of researchers and policymakers due to the emphasis in many studies on GTI’s critical role in ensuring and improving environmental quality. The growing body of research on the factors influencing the development of GTI has emphasized the significance of environmental regulation as an important driving force behind GTI. The relationship between environmental taxes and GTI was established by the use of environmental regulatory measures as incentives (Jaffe et al. 2002; Heyes and Kapur 2011; Wang and Yu 2021). Environmental regulation promotes GTI by encouraging firms to improve their global competencies and innovative capacities (Porter 1991; Porter and Linde 1995). Some studies at the national and regional levels contend that environmental regulation encourages GTI (Li and Wu 2017; Ahmed 2020; Liu and Zhao 2020; Shang et al. 2022; Wang et al. 2023) because it has a positive spillover effect on GTI, which improves GTI. In a study on OECD countries, Behera and Sethi (2022) also argue that environmental regulation in the form of environmental taxes has a significant positive impact on GTI by encouraging the economy to support GTI. Furthermore, Rubashkina et al. (2015), Zhou and Tang (2021), and Mao et al. (2022) discovered that environmental regulation encourages companies to engage in green activities and that such regulations enhance GTI. On the other hand, some studies argue that environmental regulations such as environmental taxes will impede GTI by increasing operating and input costs, reducing production with reduced efficiency, and creating a crowding-out effect (Conrad and Wastl 1995; Cinar and Yilmazer 2021; Guo et al. 2018a, b; Wei and Zhang 2020; Fang and Shao 2022). Besides, some research has established that the relationship between environmental regulation and GTI is U-shaped (Wang and Shen 2016; Ouyang et al. 2020; Song et al. 2020; Yang et al. 2020; Ai et al. 2021; Xu et al. 2021a,b; Wang and Yu 2021).

Despite the importance of determining whether and how green investments might help and impact the development of GTIs, there has been little attention dedicated to green investments as a determinant of GTIs, and there is a shortage of empirical research in this field. Among the few studies on the topic, Castellani et al. (2022) and Amendolagine et al. (2021) find that green FDI enhances GTI.

Given its importance, recent studies using different methods have been conducted to examine the factors that influence GTI. Applying a dynamic panel data model, Jin et al. (2018) have focused on the relationship between GTI and energy consumption in 28 provinces in China. The empirical results indicate that GTI increases energy consumption in the short run, but energy consumption has no significant effect on GTI. Energy consumption, on the other hand, is positively and bilaterally associated with GTI in the long run. They argue that technological innovation, as indicated in the existing literature, is unlikely to reduce energy usage. Yang et al. (2019) examine and evaluate the factors that influence energy technology innovation in China from the perspectives of fossil fuels and renewable energy sources using the generalized method of moment (GMM). First, the study discovered that the price of energy influences the development of fossil fuel technologies more than that of renewable energy technologies, indicating that China’s current energy prices are significantly below their ideal levels and that the advancement of renewable energy technology requires the support of a price mechanism. Second, public policy support has had a big impact on the development of these two categories of energy technology innovation. The accumulation of energy technology developments will support the vertical spillover effect of knowledge and will speed up the development of energy technology.

Cinar and Yilmazer (2021) use the Westerlund cointegration test, which can be applied to nonlinear series, as well as contemporary pooled mean group (PMG) and common correlated effects (CCE) estimators to analyze the relationship between investments in green energy technologies and GTI, energy prices, and environmental policies for Brazil, Russia, India, China, South Africa, and Turkey. The findings indicate that GTI is positively affected over the long term by factors such as the severity of environmental restrictions, government funding of R&D expenses, electricity prices, and the overall number of patent registrations. However, the GTI is reduced when the government promotes the use of fossil fuels, when environmental taxes are increased, and when electricity use increases. Lv et al. (2021) evaluate GTI and financial development in 30 Chinese provinces using financial structure, financial scale, and financial efficiency as metrics. The results indicate that financial structure is supportive of the development of GTI, while financial scale and financial efficiency have an adverse effect on GTI. In a very recent study, Sharif et al. (2023) examine the impact of renewable energy supply, green energy investment, environmental taxes, and economic growth on GTI in six Association of Southeast Asian Nations (ASEAN-6) countries using the Westerlund and Edgerton cointegration test and a robust CS-ARDL method. According to the results, green energy and green investment have a positive impact on GTI in both the long and short run, with the benefit being stronger in the long run. The findings also confirm the advantages of economic expansion and environmental taxation for the advancement of GTI. They argue that in order to encourage GTI in the ASEAN-6 countries, regulatory policies that support a continual increase in the percentage of renewable energy supply and investments in the agenda of environmental technological progress must be included. Bi et al. (2023) research the impact of China’s pilot Free Trade Area policy on GTI. Results demonstrate that by enhancing the marketization process and boosting the gathering of individuals with creative skills, the pilot FTZ policy promotes the growth of GTI. According to Cai et al. (2023), China’s high-quality economic development and defense science and technology innovation have a generally consistent relationship from 2010 to 2019.

Research on the determinants of GTI is very fresh and limited, despite the fact that studies examining the relationship between GTI and environmental pollution have consistently emphasized the important role of GTI in achieving and maintaining environmental quality. As a result, the literature on this subject has to be developed. We hope to contribute to this emerging topic by focusing on the determinants of GTI advances for EU countries to motivate them to accelerate their road to net-zero emissions. Additionally, the results of the limited studies on the factors that influence GTI for various nations, country groups, and regions are inconsistent. As a result, there is no consensus among researchers studying this issue. This could be due to variations in methodology, variable selection, data type, and sample size. Furthermore, given that every country differs in terms of the adoption and execution of policies and level of development, country heterogeneity may be a significant element. Therefore, a study of this kind is absolutely necessary. We think that the determinants of GTI in the 15 EU countries, which demand comprehensive and well-planned strategies to address urgent sustainability-related challenges, are examined in this research, which was conducted with an interdisciplinary approach. In this regard, the study will help fill the gap in the body of literature on this issue and advance it. The research makes significant contributions to the literature in five key areas. First and foremost, while the present research is one of the first to empirically investigate the determinants of GTI, particularly for the EU sample, it also intends to contribute to enhancing the development of the limited literature. Second, the analysis discusses the most important new environmental indicators, which include green growth, ecological footprint, and green foreign direct investments, in addition to tools like environmental taxes, renewable energy, and financial developments that are frequently discussed in the literature. Third, EU countries have not been investigated in earlier research. Fourth, the green patent data represents GTI. Green patents can reflect changes in the industry and provide a real indicator of R&D expenditures. Environmental efforts associated with innovation can indicate both quantity and quality. Finally, to achieve robust estimates, advanced econometric techniques such as the cross-sectional dependence test, PANIC unit root test, homogeneity test, and Durbin-Hausman cointegration method were applied. Furthermore, the MM-QR approach was utilized to assess the influence of the selected variables.

## Dataset and methodology

This section of the study begins with an introduction to the dataset before discussing the econometric approach used to conduct the analysis.

### Dataset

The objective of the research is to identify the variables that affect environmental technology patents for the EU region’s countries. According to the availability of data during the relevant time period, 15 countries (Austria, Belgium, Czechia, Denmark, Finland, France, Germany, Greece, Italy, Luxembourg, Poland, Portugal, Spain, Sweden, and Croatia) were chosen for this purpose. The variables compiled at annual frequency for the period 2003–2018 are introduced. These countries were chosen because of the high and low application rates for environmental technology patent applications among the EU countries. The data range and the chosen country sample are the study’s primary limitations for the previously mentioned reasons. The primary constraint in the analytical dimension is the variables included in the model within the scope of the research hypothesis. The purpose of the constraints in this case is to produce shared data. Furthermore, the variable constraint was established within the parameters of the existing literature. The data used in the analyses were obtained from the databases of institutions such as the OECD, World Bank, and UNCTAD and were therefore considered reliable secondary data.

The method’s success in detecting the variables influencing environmental technology patents when heterogeneous groups were taken into consideration was crucial in selecting the target country group. This is because, according to Aghion et al. (2022), there are a lot of environmental technology patents in EU countries, and the methodology used makes it possible to compare quantiles made by categorizing heterogeneous groups among themselves, which is a significant advantage. This will aid in determining whether the determinants of environmental technology patents differ among quantiles. Table 1 includes the variables used in the study, as well as their explanations.

**Table 1:** Data, explanation, and source

Variables	Defination	Source
Green technology ınnovation (GTI)	Patents on enviroment technologies	OECD
Renewable energy (RE)	Renewable energy consumption (% of total final energy consumption)	World Bank
Ecological footprint (EF)	Ecological footprint	Global Footprint Network
Green foreign direct ınvestment (LNGFDI)	Green FDI (value of announced greenfield FDI projects, by destination)	UNCTAD
Green growth (GG)	Green Growth, production-based CO_2_ productivity ( GDP per unit of energy-related CO_2_ emissions information on item)	OECD
Financial development (LNFD)	Domestic credit to private sector (% of GDP)	World Bank
Environmental tax (ET)	Tax revenue (% of GDP)	OECD

LN, which is at the beginning of the variables listed, represents the logarithmic transformation. Equation (1) presents the model built to accomplish the study’s aim based on all of this information.

1 $${{\mathrm{GTI}}}_{{\mathrm{it}}}= {\beta }_{0}+ {\beta }_{1}{{\mathrm{RE}}}_{{\mathrm{it}}}+{{\beta }_{2}{\mathrm{EF}}}_{{\mathrm{it}}}+ {\beta }_{3 }{{\mathrm{LNGFDI}}}_{{\mathrm{it}}}+ {\beta }_{4 }{{\mathrm{GG}}}_{{\mathrm{it}}} + {\beta }_{5 }{{\mathrm{LNFD}}}_{{\mathrm{it}}}+{\beta }_{6 }{{\mathrm{ET}}}_{{\mathrm{it}}}+ {\varepsilon }_{{\mathrm{it}}}$$

In Eq. (1), *t* denotes the period of analysis in years, while *t* = 2003,….., 2018, *T* and *i* denote the cross (countries), *i* = 1,….,15, *N*, and *ε* is the error term, represents the constant term, and it determines how the constant varies over time. The coefficients capture the change in the horizontal cross-sections over time.

The independent variables in the model with GTI as the dependent variable are EF, LNGFDI, and GG, whereas LNFD and ET are included as control variables. Furthermore, the impact of the control variables that will be included in the model is investigated in order to ensure robustness and produce better results.

The variables in the model were chosen by using studies from the literature (Sharif et al. 2023; Zeraibi et al. 2023; Lv et al. 2021; Behera and Sethi 2022; Luo et al., 2021; Huang et al. 2022; Yang et al. 2022; Javed et al. 2023; Mensah et al., 2019; Suki et al., 2022; Castellani et al. 2022).

The variable used to represent the GG is selected when the studies in the literature are evaluated based on the data availability criterion for the country group at the relevant time, in accordance with Wei et al. (2023) and Danish and Ulucak (2020).

The OECD’s green growth variable, which combines resource and environmental efficiency, aims to evaluate how economic growth lessens environmental harm through more effective use of natural resources (https://stats.oecd.org/Index.aspx?DataSetCode=EAMFP).

### Methodology and empirical results

The impact of renewable energy, ecological footprint, green FDI, green growth, financial development, and environmental taxation on GTI is explored for 15 EU countries using panel data analytic methodologies. As a result, this section introduces the econometric approaches utilized in the study to determine the relationship between the variables. First, a graphical analysis and the descriptive statistics for the variables are presented in this study. Then, Breusch-Pagan’s (1980) CD_lm1_ and Pesaran et al.’s (2008) LM_adj_ tests are applied to test for horizontal cross-section dependence.

The PANIC unit root test developed by Bai and Ng (2004, 2010) is used to investigate the variable stationarity levels. Pesaran and Yagamata (2008) devised the homogeneity test to determine whether the slope coefficients differ between units. The Durbin-Hausman cointegration test, developed by Westerlund (2008), was used to determine if there was a cointegration relationship between the variables. Finally, the MM-QR model developed by Machado and Silva (2019), which provides more comprehensive results, is estimated too.

#### Basic statistics and graph analysis

Table 2 demonstrates descriptive statistics that provide a priori information about the variables.

**Table 2:** Basic statistical tests for variables

Variables	GTI	EF	ET	GG	LNFD	LNGFDI	RE
Mean	12.21046	6.127725	2.688417	5.694542	4.447276	8.066251	17.37912
Median	12.06000	5.612458	2.530000	5.375000	4.485405	8.049939	13.56500
Maximum	25.83000	17.72611	5.100000	14.97000	5.304591	10.44520	51.91000
Minimum	3.470000	3.548612	1.580000	2.140000	2.590311	4.519503	1.280000
Std. Dev	3.683523	2.509035	0.692629	2.078287	0.428688	1.182355	12.06600
Skewness	0.858331	2.523891	1.154946	1.264574	− 0.628532	− 0.352877	1.090462
Kurtosis	4.720105	9.739703	4.210829	5.948925	4.237221	2.748681	3.323304
Jarque–Bera	59.05689	709.0370	68.01708	150.9275	31.10926	5.612511	48.60957
Probability	0.000000	0.000000	0.000000	0.000000	0.000000	0.060431	0.000000
Sum	2930.510	1470.654	645.2200	1366.690	1067.346	1935.900	4170.990
Sum Sq.Dev	3242.834	1504.567	114.6566	1032.307	43.92175	334.1134	34,795.60
Observations	240	240	240	240	240	240	240

The variables have a normal distribution since the mean and median values are close to one another. The variables in Table 2 have a normal distribution, and analysis of the data shows that the mean and median values are quite close to one another. It is also determined whether the variables have a normal distribution by examining the skewness and kurtosis results close to 0 and 3 (Cain et al. 2017).

When the kurtosis value is bigger than 3, the series is said to be pointed; when it is lower, the series is said to be kurtosis. A skewness value equal to zero indicates a normal distribution. A value greater than zero indicates that the series is positively (left) skewed, while a value less than zero indicates that the series is negatively (right) skewed (Kapusuzoğlu and Karan, 2010: 61–62).

Table 2 indicates that the values are quite distinct from 0 and 3. Accordingly, the values of the variables GTI, EF, ET, GG, and RE are left skewed since they are greater than zero, while the values of the variables LNFD and LNGFDI are right skewed since they are less than zero.

When we look at the kurtosis values, LNGFDI is flattened as its value is less than 3, while all other variables are pointed as their values are greater than 3. The LNGFDI is statistically non-normally distributed at the 10% significance level, and other variables are at the 1% significance level, according to the results of the Jarque–Bera test.

The variable graphs are shown in Fig. 1.

**Fig. 2:**
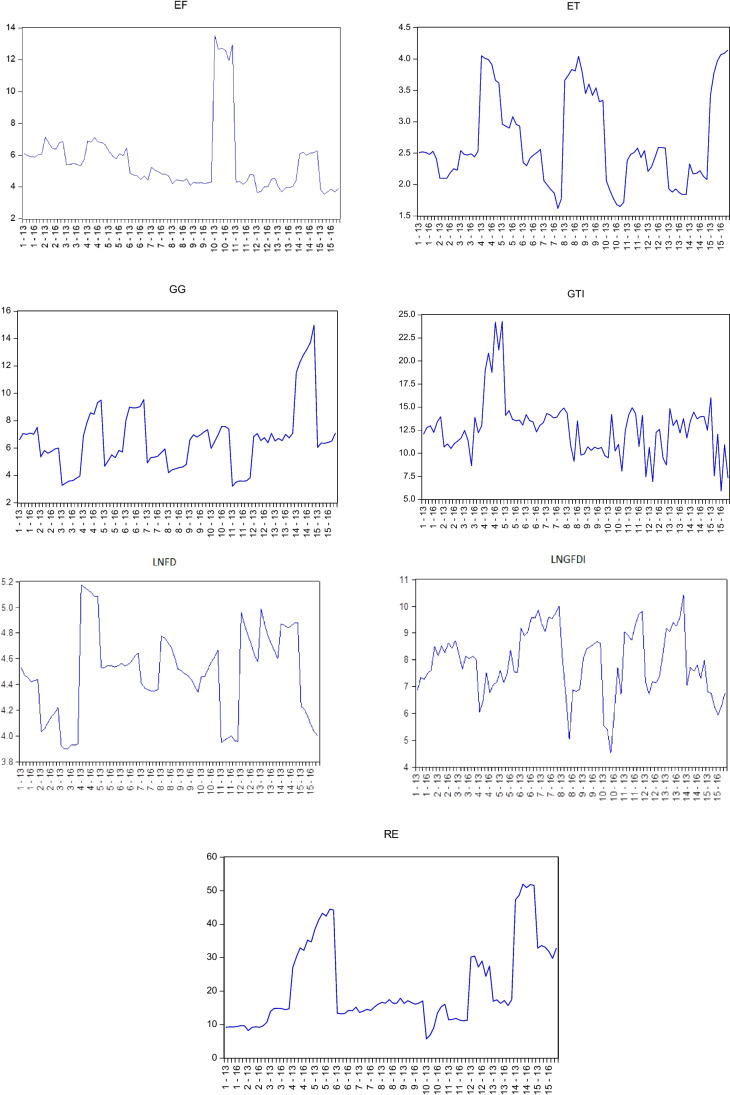
Graphical representation of variables

The GTI variable has the highest value in Denmark and the lowest value in Croatia, according to the evaluation of the variables in Fig. 2. Luxembourg and Poland have the highest values for the EF variable. In Italy, Croatia, Sweden, and Denmark, ET was comparatively higher. Sweden and Denmark have the highest rates of GG and LNFD, respectively. Poland and Belgium have the lowest LNFD values. France and Sweden were found to have the highest RE rates.

#### Cross-sectional dependence test

In panel data analysis, the variables are first examined for horizontal cross-section dependence.

This is because the tests to be used in panel data analyses are divided into first-generation tests that do not take horizontal cross-section dependence into account and second-generation tests that do. First-generation tests assume that the error terms of the horizontal cross-sections of the panel are independent and that a shock in one cross-section does not influence the others. Therefore, using first-generation tests will produce biased results in cases of horizontal cross-section dependence.

The cross-sectional dependence is examined using the Breusch-Pagan (1980) CDlm1 test and the Pesaran et al. (2008) (LM_adj_) test since the dimension of time is greater than the cross-sectional dimension (T > N). The findings are shown in Table 3.

**Table 3:** Results of cross-section dependence test

Variables	CD tests	CD_lm1_ (BP, 1980)	CD_lm2_ (Pesaran, 2004)	CD (Pesaran, 2004)	LM_adj_ (Pesaran et al., 2008)
GTI	*T* statistic	563.7069*	31.65378*	19.65618*	31.15378*
	Probability	0.0000	0.0000	0.0000	0.0000
EF	*T* statistic	734.0021*	43.40527*	24.99608*	42.90527*
	Probability	0.0000	0.0000	0.0000	0.0000
ET	*T* statistic	524.6860*	28.96109*	7.708990*	28.46109*
	Probability	0.0000	0.0000	0.0000	0.0000
GG	*T* statistic	1454.752*	93.14171*	38.05678*	92.64171*
	Probability	0.0000	0.0000	0.0000	0.0000
LNGFDI	*T* statistic	270.5064*	11.42103*	12.74196*	10.92103*
	Probability	0.0000	0.0000	0.0000	0.0000
LNFD	*T* statistic	730.9333*	43.19350*	13.29090*	42.69350*
	Probability	0.0000	0.0000	0.0000	0.0000
RE	*T* statistic	1358.365*	86.49042*	36.68353*	85.99042*
	Probability	0.0000	0.0000	0.0000	0.0000

*, **, and *** indicate that the coefficients are significant at 1%, 5%, and 10% levels, respectively

Table 3 shows that there is horizontal cross-section dependence across cross-sections and that the cross-section test statistic for all variables suggests that the null hypothesis Ho is rejected at the 1% significance level. This implies that a shock in one country will also affect the others.

Decision-makers should take this into consideration because it is clear that their actions will have an impact on other countries that are trying to improve environmental quality. On the other hand, the necessity of evaluating decisions on environmental processes on a global scale may allow countries to be affected by each other, which is an advantage in the decisions to be taken.

#### Unit root test

The non-stationarity of variables leads to the problem of spurious regression. Therefore, unit root tests are applied to determine the stationary levels of the variables. Thus, the method by which the model established to determine the relationship between variables will be estimated is also determined. Panel unit root tests are divided into two categories: first generation and second generation.

The existence of horizontal cross-section dependence requires the unit root tests of the variables to be investigated with second-generation unit root tests that take this situation into account.

The PANIC unit root test, one of the second-generation unit root tests, is addressed in this section of the study. The test developed by Bai and Ng (2004, 2010) examines the common factor and idiosyncratic components separately when investigating the presence of a unit root.

PANIC tests based on principal component analysis are presented in (2).

2 $${X}_{it}={D}_{it}+{{\lambda }{\prime}}_{i}{F}_{t}+{e}_{it}$$

${D}_{it}$ denotes the deterministic component, whereas ${F}_{t}$ and ${{\lambda }{\prime}}_{i}$ indicate the vector of common factors and vector of factor loadings, respectively. If *Ft* does not contain at least one general factor of the vector *Ft* or ${e}_{it}$ does not contain a stationary process, then ${X}_{it}$ is said to be non-stationary. There is no evidence that these two terms contain similar characteristics.

Therefore, one may follow a stationary process while the other may not.

Because it enables testing for the presence of a unit root in the residuals when the unit root in the components is denied, it is crucial in this test that the stationarity of the overall components and the residuals may be checked separately. For the stationarity of the residuals, PANIC test statistics *P*_a_ and *P*_b_ are used. The Augmented Dickey-Fuller (ADF) test statistics examining the individual stationarity of are constructed from *p* values. ADF test results with constant are denoted by *P*_a_, while findings with constant and trend are denoted by *P*_b_.

Additionally, Stock (1990) developed the panel aversion of the modified Sargan and Bhargava (1983) (PMSB) test when an ${e}_{it}$ autocorrelation issue is present. It is determined that the variable is unit rooted if any of the *P*_a_, *P*_b_, and PMSB statistics are unit rooted. The null hypothesis H_0_ of the test states that the variable has a unit root, while the alternative hypothesis H_A_ states that the variable does not contain units. Table 4 provides the results of the PANIC unit root test.

**Table 4:** Results of PANIC unit root test

Variables	Statistical values	Constant			Constant and trend		
		Pa	Pb	PSMB	Pa	Pb	PSMB
GTI	Test statistics	− 0.678	− 0.688	− 0.233	0.804	0.938	1.117
	Probability value	0.2488	0.2456	0.5922	0.7892	0.8258	0.8681
**∆GTI**	Test statistics	− 14.132*	− 5.511*	− 1.84*	− 11.937*	− 7.937*	− 2.202**
	Probability value	0.0000	0.0000	0.0329	0.0000	0.0000	0.0138
EF	Test statistics	− 1.715**	− 1.257	− 1.039	− 1.14	− 1.021	− 0.731
	Probability value	0.0432	0.1044	0.1494	0.1271	0.1537	0.2322
**∆EF**	Test statistics	− 2.362*	− 1.652**	− 1.949***	− 1.611**	− 1.337***	− 1.803***
	Probability value	0.0091	0.0492	0.0812	0.0536	0.0906	0.0914
ET	Test statistics	− 1.517***	− 1.201	− 0.826	− 0.435	− 0.419	− 0.26
	Probability value	0.0647	0.1149	0.2044	0.3318	0.3377	0.3975
**∆ET**	Test statistics	− 6.041*	− 3.409*	− 1.679**	− 1.984**	− 1.626**	− 1.985***
	Probability value	0.0000	0.0003	0.0466	0.0236	0.052	0.0907
GG	Test statistics	− 3.182*	− 1.901**	− 1.408***	− 0.746	− 0.677	− 0.471
	Probability value	0.007	0.0287	0.0796	0.228	0.2491	0.3189
**∆GG**	Test statistics	− 9.289*	− 3.501*	− 2.243***	− 2.562**	− 1.891**	− 2.022
	Probability value	0.0000	0.0002	0.0845	0.0052	0.0293	0.0834
LNGFDI	Test statistics	− 6.107*	− 3.419*	− 1.906**	− 1.559**	− 1.35***	− 0.925
	Probability value	0.0000	0.0003	0.0283	0.0595	0.0885	0.1774
**∆LNGFDI**	Test statistics	− 20.618*	− 7.417*	− 2.435*	− 6.786*	− 4.516*	− 1.899**
	Probability value	0.0000	0.0000	0.0074	0.0000	0.0000	0.0288
**LNFD**	Test statistics	2.144	2.023	0.328	3.256	4.2154	1.4787
	Probability value	0.984	0.9785	0.6285	0.7851	0.8823	0.5488
**∆LNFD**	Test statistics	− 0.394	− 0.365	− 0.092	− 2.199**	− 1.785**	− 2.106***
	Probability value	0.347	0.3574	0.4634	0.0139	0.0371	0.0944
**RE**	Test statistics	− 0.264	− 0.208	− 0.55	0.513	0.563	0.619
	Test statistics	0.3958	0.4176	0.2912	0.6959	0.7132	0.7319
**∆RE**	Probability value	− 10.779*	− 5.114*	− 2.087**	− 5.068*	− 3.589*	− 1.722**
	Test statistics	0.0000	0.0000	0.0185	0.0000	0.0002	0.0426

*, **, and *** indicate that the coefficients are significant at 1%, 5%, and 10% levels, respectively

In the PANIC unit root test, if even one of the probability values *P*_a_, *P*_b_, and PSMB is not significant, the variable has a unit root. When the results in Table 4 are analyzed, it is observed that while the GG variable is stationary at level in the model with constant, it has a unit root at level in the model with constant and trend. In addition, the degree of stationarity of GG strengthens when its difference is taken.

While LNGFDI is stationary in the model with constant, it is unit rooted in the model with constant and trend. When the difference is taken, the degree of stationarity strengthens in the model with constant, while it becomes stationary in the model with constant and trend.

LNFD is found to be stationary when the difference is taken in the model with constant and trend. When the other variables are differenced, they are stationary both in the model with constant and in the model with constant and trend.

The fact that the variables are stationary when differenced indicates that the shock to the series persists in the long run. Since differencing non-stationary series eliminates the possibility of a long-run relationship, cointegration analysis is preferred. Because of this, cointegration tests can be used to find out whether the variables move together in the long time. Furthermore, finding the dependent variable stationary when the difference is taken allows the Durbin-Hausman cointegration test to be applied to determine the long-run relationship between the variables.

#### Homogeneity test

The homogeneity of the slope coefficients in the panel cointegration equation is investigated using the delta (Δ̃) and adjusted delta (Δ̃ *adj*) tests developed by Pesaran and Yagamata (2008).

If the homogeneity test findings show that the slope coefficients are heterogeneous, the long-run relationship between the variables is assessed using the second-generation cointegration test, which takes this situation into consideration. In the homogeneity test, the main hypothesis H_0_ states that the slope coefficients are homogeneous, and the alternative hypothesis H_A_ states that the slope coefficients are heterogeneous. Table 5 shows the variables’ homogeneity test results.

**Table 5:** Results of the homogeneity test

Test İstatistikleri	Test İstatistiği	Olasılık Değeri
Δ̃	4.109*	0.000
Δ̃ *adj*	6.213*	0.000

* indicates statistical significance level of 1%

Table 5 reveals that the null hypothesis H_0_ is statistically rejected at the 1% level of significance for both test statistics and that the coefficients are different. The model’s heterogeneity indicates that the cross-sectional units have different characteristics.

#### Durbin-Hausman cointegration test results

The Durbin-Hausman cointegration test, developed by Westerlund (2008), is utilized in this study to examine the long-term relationship between the variables while taking into account horizontal cross-section dependence. In order to apply the method, the dependent variable must be stationary at first difference, while the independent variables can be stationary at level or at first difference (Westerlund, 2008: 205).

The Durbin-Hausman cointegration test includes test results that allow for homogeneity and heterogeneity. Accordingly, the Durbin-H panel group statistic gives the results taking heterogeneity into account, while the Durbin-H panel test statistic gives the test results taking homogeneity into account. The null hypothesis H_0_ of the test is that there is no cointegration relationship for all units, while the alternative hypothesis H_A_ is that there is a cointegration relationship for some units.

Table 6 shows the outcomes of the Durbin-Hausman cointegration test. Since the homogeneity test results reveal that the coefficients are heterogeneous, the Durbin-H Group test statistic results will be taken into account in the cointegration test.

**Table 6:** Durbin-Hausman cointegration test results

Test statistics	Statistics value	Probability value
Durbin-H Group statistic	1456.865	0.000
Durbin-H Panel statistic	16.055	0.000

* indicates statistical significance level of 1%

Table 6 demonstrates that the Durbin-H Group statistic statistically rejects the H_0_ hypothesis at the 1% significance level and finds that the variables have a long-run relationship. According to cointegration analysis, it is stated that even if the series of variables are non-stationary, a stationary combination of these series may exist, and if so, it can be determined (Tarı, 2010: 415). The fact that the effects of actions aimed at improving environmental quality will reveal themselves in the long run also reveals the importance of determining the long-run relationship between variables. As a result, the discovery that environmental technology patents and their determinants move together in the long time has been revealed to be a theoretical instance of the long-run relationship between these variables.

#### Method of Moment Quantile-Regression (MM-QR) estimation model

In this study, the effect of the independent variables on the dependent variable is investigated with the MM-QR estimation method developed by Machado and Silva (2019) by taking into account the distribution function along different quantiles. MM-QR is performed by taking into account the distribution function along different quantiles, taking into account the presence of fixed effects.

The MM-QR estimation method developed by Machado and Silva (2019) has the advantage that it can be applied to nonlinear models and is computationally much simpler, especially in models with multiple endogenous variables (Hieu and Mai 2023:584). The estimation method develops an estimator of the conditional quantiles obtained by combining the estimates of the location and scale functions determined by appropriately defined conditional expectations (Machado and Silva 2019).

So, conditional quantile *Q*(τ/*X*) related to the “locational-scale alternate model” is developed as under Alwehab (2022: 224).

3 $${Y}_{it}={\alpha }_{i}+{X}_{it}\beta +\left({\delta }_{i}+{Z}_{it}\lambda \right){U}_{it}$$

In Eq. (3), the probability is represented by *P* $\left\{{\delta }_{i}+{Z}_{it}\lambda >0\right\}=1$ while the unknown parameters are represented by α, β, λ, and δ. Equation (4) represents the transformation of the components, and $Z$ produces a differentiable vector $k$ of known transformations of the $X$ components.

4 $${Z}_{l}={Z}_{l}\left(X\right),l=1,\dots ,k$$

${U}_{it}$is independent and identically distributed across units and time. It is also statistically independent of${X}_{it}$ and normalized to satisfy the moment condition in the method.

${U}_{it}$ and ${X}_{it}$ are similarly distributed beyond time-period (*t*) and individual (*i*). For any fixed ${U}_{it}$, ${X}_{it}$ is normally distributed and time-independent (*t*). As stated by Machado and Santos Silva (2019), ${U}_{it}$ is the standardized momentum conditions orthogonal to ${X}_{it}$ (Adebayo et al. 2022: 32,291). This is illustrated as follows:

5 $${Q}_{y}\left(\tau \left|X\right.\right)=\left({\propto }_{i}+{\delta }_{i}q\left(\tau \right)\right)+{{X}_{it}}{\prime}\beta +{{Z}_{it}}{\prime}\gamma \left(\tau \right)$$

In Eq. (5), the scalar coefficient $\left({\propto }_{i}+{\delta }_{i}q\left(\tau \right)\right)$ represents the quantile-$\tau$ constant effect for individual $i$ or the distributional effect in $\tau$. The distributional effect differs from the fixed effect as there is generally no position shift. That is, it shows the effect of individual characteristics that do not change over time. ${\int }_{0}^{1}q\left(\tau \right)d\tau$ reveals that ${\propto }_{i}$ can be expressed as the average effect for individual $i$. Accordingly, $\tau$ th sample quantile is calculated by solving the optimization problem in (6) (Machado and Silva 2019).

6 $$\underset{{\mathrm{q}}}{{\mathrm{min}}}\sum_{i}\sum_{i}{\rho }_{\tau }\left({\widehat{R}}_{it}-\left({\widehat{\delta }}_{i}+{{Z}_{it}}{\prime}\gamma \right)q\right)$$

In Eq. (6), ${\rho }_{\tau }\left(A\right)=\left(\tau -1\right){\mathrm{AI}}\left\{A\le 0\right\}+T{\mathrm{AI}}\left\{A>0\right\}$ denotes a control function (Adebayo et al. 2022: 32,291).

Table 7 shows the results of the MM-QR estimation, which will offer information on the coefficient estimate and direction of the impact of green FDI, green growth, environmental taxes, and renewable energy on environmental technology patents based on heterogeneous groupings.

**Table 7:** MM-QR estimation results

Variables	Location	Scale	Quantiles								
			0.10	0.20	0.30	0.40	0.50	0.60	0.70	0.80	0.90
RE	0.0135908	0.006837	0.0028821	0.0058324	0.0089922	0.0109758	0.0138158	0.0157916	0.0187887	0.0212244	0.0242242
GG	− 0.2068517***	− 0.2273875*	0.1493012	0.0511774	− 0.0539108	− 0.1198816	− 0.2143362***	− 0.280047***	− 0.379726*	− 0.460735*	− 0.5605025*
EF	0.0835685	0.1097809	− 0.0883794	− 0.0410059	0.0097299	0.0415801	0.087182	0.1189067	0.167031	0.2061415	0.2543085***
LNGFDI	0.0105311	0.0370117	− 0.0474396	− 0.0314681	− 0.014363	− 0.003625	0.0117493	0.022445	0.0386697	0.0518554	0.0680945
LNFD	3.78693*	1.014428*	2.19805*	2.635803*	3.104625*	3.398936*	3.82032*	4.113471*	4.558162*	4.919561*	5.364647*
ET	0.0860491	0.8826672*	− 1.296457**	− 0.9155618***	− 0.507633	− 0.2515492	0.1151025	0.370177	0.7571085	1.071567***	1.458842**
*c*	− 4.517701	− 4.015946	1.772402	0.0394124	− 1.816576	− 2.981702	− 4.649888	− 5.810422	− 7.570877	− 9.001597	− 10.76361

When the results in Table 7 are analyzed, it is determined that the effect of GG on GTI is statistically significant and negative in countries in the medium (0.4, 0.5, and 0.6) and high (0.7, 0.8, and 0.9) quantile groups. This negative relationship is in the opposite direction of the expectation. This is because GG and GTI are complementary elements and are expected to contribute to environmental sustainability by acting together. The fact that the GG coefficient is negative in the country groups with medium and high quantiles initially shows that green growth inhibits the advancement of green technologies. However, the fact that GG is represented in the study by resource efficiency that reduces CO_2_ emissions in production processes may mean that these countries in the EU group have not been able to put forward GTI studies to achieve such efficiency. In other words, it may have demonstrated that CO_2_-reducing technologies in the production process have not yet supported the increase in GTI that would have a positive impact on environmental quality.

For EU countries, it is possible that CO_2_ efficiency practices in the production process have not yet been included in the environmentally friendly production process due to a number of problems (high cost, reluctance of individuals or organizations towards such investments, limitations in the application of green technology for production). Therefore, practices to reduce the costs of green technologies can support an environmentally friendly production process by contributing to green technology. Finally, another conclusion is that EU countries need to accelerate such investments.

The effect of EF on GTI is statistically significant and positive for countries in the 0.9 quantile group. An increase in EF means a decrease in environmental quality. Therefore, an increase in GTI that emphasizes practices to improve environmental quality for a sustainable life will be triggered.

The effect of LNFD on GTI is statistically significant and positive in all quantiles. However, it is also observed that the positive effect increases as the quantile level increases. This result is similar to that of Lv et al. (2021). The financial resources needed for GTI’s expansion and development. The LNFD will make it easier for investors to adopt green technology by facilitating financing for projects including green technology in support of the GTI. Investors can be encouraged to realize eco-friendly production in this way. The finding of an increasing effect in the high-quantile ever group provides support for this conclusion.

Finally, ET has a statistically significant negative and positive effect on GTI for countries in the low and high quantile groups, respectively. The results obtained were similar to Shang et al. (2022), Ahmed (2020), and Sharif et al. (2023) for the quantitative group where ET was high. The negative effect of ET on GTI for countries in the low-quantile group is similar to Cinar and Yilmazer (2021).

Due to the expenses they place on businesses in the process of controlling waste and pollution, environmental taxes push enterprises to create green technologies to enhance their manufacturing processes. Therefore, in order to lower this cost, countries with high ET invest in environmentally friendly production technology, which raises the number of green patent applications. On the other hand, the negative impact of low ET on GTI reveals that in the group of countries with low environmental taxation, the low cost of this cost does not encourage technological steps towards environmental improvement.

## Discussion

GTI is an effective driver in achieving zero emissions, environmental quality, and green transformation with green growth, enhancing the production of goods, and boosting energy efficiency. GTI may mitigate non-renewable energy consumption while increasing renewables and energy efficiency. Green growth is also an efficient way of reducing energy consumption and environmental pollution and is considered by many as a way to ensure environmental quality. GTI is an effective tool that helps to improve the quality of the environment. In order to embark on this program, several policies have been adopted globally. Thus, by removing the detrimental effects of economic activity on environmental quality, developing public policies for a sustainable environment and a green future depends on an understanding of the factors affecting GTI. EU countries create rules, such as the EU Emission Trading System and recently the Net-Zero Industry Act, to stimulate low-carbon technology advancement and accelerate the transition to zero emissions in order to establish a low-carbon economy, taking the lead in the battle against climate change. Recognizing the urgency of the transition to low-carbon living for EU countries, the present research offers a novel investigation of possible drivers of green technological developments for EU countries from 2003 to 2018, including environmental taxes, renewable energy, financial development, greenfield FDI, ecological footprint, and green growth.

To evaluate the parameters influencing environmental technology patents in selected EU countries, basic statistical calculations and graphical analyses were performed first. The LM_adj_ test was used to examine the horizontal cross-sectional dependency among the variables, and it was concluded at the 1% significant level that there is horizontal cross-sectional dependence between all cross-sections of all variables included in the model. The PANIC unit root test, one of the second generation unit root tests, was used to analyze the variables’ stationarity, and the findings showed that the variables were stationary at the *I*(*I*) level. According to the Delta test results, slope coefficients vary between units. In other words, it is concluded that the variables are heterogeneous. The Durbin-Hausman cointegration test, used in the study to identify the factors influencing environmental technology patents, established that the variables in the model have a long-term relationship. The result of this study is in agreement with expectations and is supported by the relevant research and theory. The effectiveness of the measures that have to be taken and the policies to be put into place to deal with environmental issues will be felt in the long run. This result also highlights the significance of determining the long-run relationship between the variables included in the model, namely, GTI, ecological footprint, greenfield FDI, green growth, financial development, and environmental tax. Finally, after determining the variables’ long-run relationship, the MM-QR estimation method was used to examine the effect of the dependent variable on the independent variables. According to the results, the effect of green growth on GTI is statistically significant and negative in countries in the medium and high quantile groups. For countries in the 0.9 quantile group, the effect of ecological footprint on GTI is statistically significant and positive. Financial development has a statistically significant, positive impact on GTI in all quantiles. However, it is also observed that when the quantile level rises, the positive impact gets stronger. Environmental tax has a statistically significant negative and positive effect on GTI for low and high quantile countries, respectively.

According to the findings, there is a long-term relationship between GTI and environmental taxes, renewable energy, financial development, greenfield FDI, ecological footprint, and green growth. This suggests that countries need to identify the factors that affect GTI for the transition to low-carbon living and manage the policies that will ensure this transition more carefully. This finding will significantly advance both the body of literature and the chosen EU country group. Additionally, we employ the unique and reliable MM-QR approach to estimate the determinants of GTI, which is another novel contribution of the study to the literature. This approach highlights heterogeneous factors necessitating primary attention within EU countries for GTI. A well-known indicator for measuring GTI is green patent data. The green patent variable is employed as the GTI. It is believed that the study’s use of green patent, a more comprehensive variable that covers environmental regulation, as a proxy for GTI makes a significant contribution to the literature. Besides, 15 countries from the EU were selected as the study’s sample country group. In research studies examining the determinants impacting GTI in the literature, this country’s group has not been examined. Given that the EU is tackling this issue with a number of action plans for the transition to low-carbon life, the effectiveness of this group of countries is important. In conclusion, this study aims to close gaps in the literature from various perspectives. In particular, the recent search for new sources to meet energy needs in the EU may also apply to other countries. Moreover, the MM-QR methodology used classifies different levels of green technology advances into low, medium, and high groups. This may contribute to the policies for all countries in this study, which is based on the EU-specific case. As a result, the present research is unique in that it investigates the determinants of GTI in countries of the EU that are combating climate change and have put critical action plans in place for achieving a low-carbon lifestyle. Furthermore, we applied advanced econometric approaches such as the cross-sectional dependence test, PANIC unit root test, homogeneity test, and Durbin-Hausman cointegration method to obtain robust results. Finally, unlike previous studies, the analysis discusses the most important new environmental indicators, such as green growth, ecological footprint, and green foreign direct investments, as well as tools such as environmental taxes, renewable energy, and financial developments that are frequently discussed in the literature.

## Conclusions

The findings from the analysis of the drivers of GTI for 15 selected EU country groups are generally consistent with the few studies available in the literature. In this regard, it is generally consistent with the studies of Ahmed (2020), Cinar and Yilmazer (2021), Lv et al. (2021), Shang et al. (2022), and Sharif et al. (2023). The variables employed in the research in the literature, the country groups chosen, and the data ranges are all different. Furthermore, the PANIC unit root test and Durbin-Hausman cointegration tests employed in the study are not commonly used in the literature; hence, the work is methodologically unique.

Based on the results of the study examining the relationship between GTI and environmental taxes, renewable energy, financial development, greenfield FDI, ecological footprint, and green growth, policymakers and new researchers can make some suggestions. Policymakers can play a supportive and developmental role towards facilitative practices in the steps to be taken in this area. In this context, the following recommendations are made for policymakers. The fact that financial development has a significant growing effect on GTI in all quantiles might be read as financial development being the most strategic factor of GTI. One of the most efficient strategies to promote GTI-related activity may be direct transfer of financial resources to firms and start-ups specializing in low-carbon technology research and development area. Monitoring processes that will provide a competitive advantage to companies and start-ups in the private sector and facilitate their access to low-cost financial resources can be one of the most effective market tools. In order to extend this facility to other companies operating in the market, it is important for financial institutions to diversify into special green funds that are attractive and supportive of green innovation. Financial development also encourages GTI by accelerating capital turnover. Prioritization of enterprises producing low-pollution technology can therefore be an effective policy tool. Financial development was found to be a very effective factor for GTI in EU countries, but greenfield FDI did not exhibit an impact in this country group. Greenfield FDI has not been found to have a significant effect on GTI. The technology spillover effect of greenfield FDI on green innovation is not yet effective. Given the importance of structural components in greenfield FDI, establishing product market regulations to incentivize low-carbon foreign investments and reducing needless regulatory barriers to such investments might be a significant initiative for technology diffusion. In particular, regulations that offer incentives for enterprises to develop green technologies to enhance their production procedures should be prioritized when enacting environmental taxes. The rise in pollution emissions and footprints in the atmosphere is one of the primary reasons for the need for environmentally friendly technologies. The study’s findings also revealed that an increase in ecological footprint raises GTI. Therefore, it is critical that countries create regulations that take the ecological footprint into account. Finally, new researchers may be encouraged to examine the impact of various variables that may have an impact on GTI. It is thought that by analyzing different country groups, new scholars can reach different conclusions across different income levels. The final piece of advice for new researchers is to conduct analyses using various statistical and econometric methods. Because the study included a group of countries, panel data analysis techniques were used in the study. Using time series analysis and new generation tests, researchers may evaluate the results of a country and develop policy recommendations.

## Data Availability

Data availability raw data used in our analysis are available as Supplementary Material.
